# Patterns of Genetic Diversity and Mating Systems in a Mass-Reared Black Soldier Fly Colony

**DOI:** 10.3390/insects12060480

**Published:** 2021-05-21

**Authors:** Lelanie Hoffmann, Kelvin L. Hull, Anandi Bierman, Rozane Badenhorst, Aletta E. Bester-van der Merwe, Clint Rhode

**Affiliations:** 1 Department of Genetics, Stellenbosch University, Private Bag X1, Matieland 7602, South Africa; hoffmann.lani@gmail.com (L.H.); 17507537@sun.ac.za (K.L.H.); aeb@sun.ac.za (A.E.B.-v.d.M.); 2 Insect Technology Group Holdings UK Ltd., 1 Farnham Road, Guildford GU2 4RG, UK; anandie@sun.ac.za (A.B.); rozanebadenhorst@gmail.com (R.B.)

**Keywords:** assortative mating, *Hermetia illucens*, insect culture, genetic differentiation, microsatellite markers, multiple paternity

## Abstract

**Simple Summary:**

With a growing human population, global climate change and diminishing natural resources, the need for sustainable agricultural systems is evident. Insect farming has been shown to be an envi-ronmentally friendly alternative to conventional livestock farming. The black soldier fly (BSF) is a species of interest because the larvae are effective bioremedial agents, feeding on organic waste and converting it to usable animal derived products that have a similar nutrient profile to conventional feedstuffs like soy and fishmeal. This creates an opportunity for renewable food production systems. Managing genetic diversity in commercial insect populations is important for the long-term viability and productivity of the colony to mitigate any negative effects of inbreeding. In this study we in-vestigated the effects population dynamics and breeding behaviour on the genetic health of a mass reared BSF colony. The results suggest significant loss in genetic diversity and rapid divergence of captive populations from ancestral wild ones. The study also gives the first evidence for multiple paternity in BSF. The results will contribute to establishing effective genetic management strategies for BSF farming, ensuring long term sustainability of a new industry

**Abstract:**

The black soldier fly (BSF), *Hermetia illucens*, is a promising candidate for the emerging insect farming industry with favourable characteristics for both bioremediation and production of animal delivered nutritive and industrial compounds. The genetic management of commercial colonies will become increasingly important for the sustainability of the industry. However, *r*-selected life history traits of insects pose challenges to conventional animal husbandry and breeding approaches. In this study, the long-term genetic effects of mass-rearing were evaluated as well as mating systems in the species to establish factors that might influence genetic diversity, and by implication fitness and productivity in commercial colonies. Population genetic parameters, based on microsatellite markers, were estimated and compared amongst two temporal wild sampling populations and four generations (F28, F48, F52, and F62) of a mass-reared colony. Furthermore, genetic relationships amongst mate pairs were evaluated and parentage analysis was performed to determine the oc-currence of preferential mate choice and multiple paternity. The mass-reared colony showed a reduction in genetic diversity and evidence for inbreeding with significant successive generational genetic differentiation from the wild progenitor population. Population-level analysis also gave the first tentative evidence of positive assortative mating and genetic polyandry in BSF. The homoge-neity of the mass-reared colony seems to result from a dual action caused by small effective popu-lation size and increased homozygosity due to positive assortative mating. However, the high ge-netic diversity in the wild and a polyandrous mating system might suggest the possible restoration of diversity in mass-reared colonies through augmentation with the wild population.

## 1. Introduction

As the global human population continues to grow, the sustainability of agricultural production and food security is becoming an increasing concern, especially considering global climate change and diminishing natural resources. To meet the growing demand for alternative sources of protein, the mass-rearing of insects has gained attention worldwide. This is particularly due to the low input costs, resource ‘lite’ nature, and high feed conversion ratios of insects [1,2]. In particular, the production of *Hermetia illucens* (L. 1758; Diptera: Stratiomyidae) (black soldier fly, BSF) has increased considerably in recent years [3,4]. The popularity of the species is due to the larvae’s comparative nutrient profile to conventional protein sources like soy and fishmeal [5,6,7] and its global, cosmopolitan distribution [8]. The species is thought to have originated in South America and has since spread to most (sub)tropical and temperate regions of the world, highlighting the BSF’s adaptability to a variety of conditions [8]. Furthermore, the larvae are efficient feeders of organic waste, creating the opportunity for circular and renewable agricultural systems, where larvae feed on agricultural waste and in turn larval products are used in animal feed and plant fertiliser production [9,10].

As the mass production of BSF is in its early phases, current research on mass-reared colonies is focused on the creation of optimal mass-rearing environments and husbandry practices. Knowledge on the impact of induced domestication on the genetic health of commercial colonies is therefore limited. Rhode et al. [11] have shown that the early stages of captive rearing of a wild-caught BSF colony can have major effects on genetic composition and phenotypic development, and place such a colony at risk of collapse. The adverse effects of captive mass-rearing and domestication are associated with the loss of genetic diversity. Genetically diverse populations generally exhibit greater fitness and robustness to environmental stressors and disturbances, such as unexpected harsh climatic conditions and disease outbreaks [12,13,14,15], which in turn ensures the high productivity of a commercial colony [14,16]. Lessons from conventional livestock and aquaculture production have shown the importance of effective management of genetic diversity during the domestication and genetic improvement of species for the long-term sustainability of production [17,18,19,20]. Managing and maintaining genetic diversity in commercial, mass-reared colonies can, however, be challenging.

During the process of domestication, the founder effect causes an initial population bottleneck, a sudden and abrupt reduction in effective population size, which enhances the loss of genetic diversity through random genetic drift [21]. The effective population size is further reduced by any selective sweeps that might occur due to the novel captive environment, further pronouncing the effects of random drift and increasing the likelihood of inbreeding. This leads to the genetic homogenisation of the population that is phenotypically expressed as reduced fitness as a consequence of inbreeding depression [14,22,23,24]. This problem is compounded in insects due to their *r*-selected life history characteristics. Briscoe et al. [25] studied the effects of captivity on genetic variation in *Drosophila melanogaster* and found that up to 62% of population genetic diversity could be lost in as little as 26 generations, and up to 86% could be lost in 56 generations.

Isolated populations suffering from severe inbreeding often evade inbreeding depression through the process of genetic purging. While this is effective in the short term, purifying selection decreases genetic diversity even more [24,26]. Genetic rescue is a method of improving fitness in a commercial population while also reintroducing genetic diversity. This is achieved by introducing immigrants from donor populations into the inbred population. Genetic rescue has shown great success in the past, even when using two inbred lines of *Drosophila melanogaster* to augment each other [27,28]. However, success is dependent on the genetic similarity between immigrants and the inbred population. The introduction of individuals that are genetically too different from the commercial colony may lead to a decrease in offspring fitness, by disrupting coadapted gene complexes or introducing maladaptive alleles. This occurrence is referred to as outbreeding depression [29,30].

The negative effects of inbreeding on fitness and productivity, as well as the risks associated with reintroducing diversity into a population, highlight the importance of managing and maintaining a healthy level of genetic diversity in mass-reared, commercial BSF colonies. As mass-rearing facilities approach optimal husbandry, beyond the initial stages of colony establishment, their focus is starting to shift from environmental optimisation to the long-term maintenance and genetic improvement of colonies.

Other than the micro-evolutionary and demographic factors discussed above, maintenance of genetic diversity in a population is also a function of the organism’s mating system. There are four potential mating systems, with varying influences on genetic diversity. Strict monogamy is rare in insects (e.g., [31,32]), but these species tend to exhibit lower effective population sizes and thus lower levels of genetic diversity in comparison to polygamous species. The three remaining mating systems are all variations of polygamy: polygyny, polyandry, and polygynandry. Polygyny refers to the mating of one male with multiple females. This mating system is common in animals with a male dominant social structure and has been postulated in several species of flies, including the stalk-eyed fly and the olive fly [33,34]. Due to small effective population sizes, polygynous populations are more likely to be genetically homogenous and susceptible to the adverse effects of inbreeding and loss of diversity [35,36]. Polyandry, where females mate with multiple males, is common in many insect species, especially eusocial insects, such as bees and ants [13,37,38]. Polyandry is typically associated with increased heterozygosity, when compared to both monogamy and polygyny [39]. The mating of both sexes with multiple mates, as seen in *Drosophila melanogaster*, for example, is known as polygynandry [40]. As all individuals have the potential to mate multiple times, this mating system is the most conducive to the maintenance of genetic diversity.

Black soldier flies exhibit lekking, a mating behaviour where males aggregate at sites known as leks, each defending their own small territory. Females then visit these lekking sites for the sole purpose of mating [41]. Fly species that show lekking behaviour include melon flies, Mediterranean fruit flies and sandflies [42,43,44]. Lekking has also been observed in other species from the genus *Hermetia* [45]. As lekking behaviour can help males to mate with multiple females, lekking insects often exhibit polygynous mating behaviour. Evidence for polyandry has also been found in selected lekking species [42,43,44,46].

Genetic diversity may also be influenced by the presence of selective mate choice. In populations exhibiting random mating, genotypic frequencies remain stable over time. However, when sexual selection occurs, equilibrium genotypic frequencies are disrupted, potentially affecting genetic diversity [47]. Negative assortative mating occurs when individuals mate with partners that are either genotypically or phenotypically dissimilar. This leads to a higher incidence of intermediate phenotypes, which increases heterozygosity [48]. When individuals mate with partners that are either genotypically or phenotypically similar, positive assortative mating occurs. This favours extreme phenotypes, thereby increasing homozygosity in the population [48]. Inbreeding is also a form of extreme positive assortative mating, as it increases homozygosity in populations through the mating of genetically related individuals. When assortative mating occurs based on phenotypic selection, changes in heterozygosity are limited to genes relating to the phenotype [49]. Inbreeding, however, increases homozygosity across the entire genome [50].

It has been observed that BSF females only produce a single viable clutch of eggs in their lifetime [51], possibly as a result of resource depletion. Adult flies do not regularly feed [52], although they can ingest liquid/semi-solid substrates [53,54,55]. Furthermore, Samayoa et al. [56] observed that females were able to mate multiple times, but due to only having a single viable egg clutch, multiple paternity could not be confirmed, and the prevailing hypothesis is that this species is genetically monogamous. In addition to this, no evidence for discriminate mating has been observed in BSF populations.

Given the interplay between genetic diversity and mating systems, this study aimed to particularly assess the maintenance and long-term trends of genetic diversity in an established, mass-reared colony of BSF, as well as to evaluate mating behaviour in a mass-reared colony via mate pair genetic comparisons and parentage analysis.

## 2. Materials and Methods

### 2.1. Sample Collection and DNA Preparation

Assessment of genetic diversity: Two sampling cohorts of 30 adult flies each were randomly selected from a wild BSF population in Durban, South Africa (29.8587° S, 31.0218° E) in 2015 (Wild_2015_) and 2018 (Wild_2018_), respectively. These cohorts represent the wild founding population of the mass-reared colony. The mass-reared colony itself was established in 2012, from an unknown number of founding wild flies. Since establishment, the colony was maintained under industry standard mass-rearing production protocols with no additional population augmentation with wild flies during latter generations. Each generation was reared discretely with no overlapping or admixture between generational cohorts. At the start of the experiment the colony was at the 28th generation. To assess long-term trends, sampling occurred approximately every 15–20 generations with one “intermediary” sampling event (F52). As such, 30 adult flies per generation were collected from generations F28, F48, F52 and F62 of a mass-reared colony under factory conditions. Each fly was stored individually in a tube containing 90% ethanol, at −20 °C. Parts of the head, thorax, and legs of individuals were removed using a sterile surgical scalpel for genomic DNA extraction. A modified version of the cetyltrimethyl ammonium bromide (CTAB) extraction method, with the addition of proteinase K to accommodate the extraction of DNA from insects, was used [57,58]. After extraction, the quantity and quality of isolated genomic DNA was evaluated using a NanoDrop^TM^ ND 1000 spectrophotometer (Thermo Fisher Scientific, Waltham, MA, USA). Working dilutions with a final concentration of 20 ng/µL were prepared and stored at −20 °C.

Assessment of mating behaviour: Samples for parentage analysis were collected from the 48th generation of the mass-reared colony. Five mating pairs were captured in copula and placed in separate containers, each containing a block for the female to oviposit on. Upon oviposition, both the male and female were collected and stored in 90% ethanol. The female was considered the known mother of the offspring, while the male was considered a candidate father. Each egg clutch was incubated and reared separately. After hatching, neonate larvae were fed a standard artificial diet for six days before being collected and stored in 90% ethanol. Twenty-five larvae per clutch were selected at random, to test for the presence of multiple paternity. To test for assortative mating, thirty additional flies were randomly collected from the source population. DNA extractions were performed as described above.

### 2.2. Genotyping

Eight microsatellite loci previously developed by Rhode et al. [11] were amplified in three multiplex PCR reactions. A final reaction volume of 10 µL included KAPA2G™ Fast Multiplex PCR Mix, 20 ng of genomic DNA and 0.8 µM of each fluorescently labelled forward primer and reverse primer. Reactions started with an initial denaturing step at 95 °C for 5 min. This was followed by 35 cycles of denaturation at 95 °C for 15 s, annealing at the annealing temperature (T_a_) for 30 s, and an extension step at 72 °C for 90 s. Reactions were concluded with a final extension at 72 °C for 30 s. Successful amplification was confirmed through visualisation via 1.5% agarose gel electrophoresis, 100 Volts for 45 min. Samples were then diluted with double-distilled water at a ratio of 3ddH_2_0: 1DNA before capillary electrophoresis at the Stellenbosch Central Analytical Facility’s DNA Sequencing Unit. Alleles were scored from chromatogram data using GeneMapper v5.0.3 (Applied Biosystems, Waltham, MA, USA) and a GeneScan™ 500 LIZ^®^ (Applied Biosystems, Waltham, MA, USA) DNA ladder standard.

### 2.3. Genetic Data Analysis

Assessment of genetic diversity: Input files for relevant software were first created using Microsatellite Toolkit v3.1 [59]. Micro-checker v2.2.3 [60] was used to check for null alleles, stuttering and allelic drop out at each locus (1000 randomisations, 95% confidence interval). The Brookfield 1 method was used to estimate null allele frequencies [61]. Exact tests were performed in Genepop on the web v4.7 [62,63] to test for Hardy–Weinberg equilibrium (HWE). Genetic diversity statistics including the polymorphic information content (PIC), number of alleles (*A_N_*), effective number of alleles (*A_E_*), observed and unbiased expected heterozygosity (*H_O_* and u*H_E_*, respectively), Shannon’s information index (*I*), and per locus *F_IS_* were calculated using GenAlEx v6.503 [64]. A mean inbreeding coefficient (*F_IS_*) for each group was determined by calculating the average of per locus *F_IS_* estimates. The allelic richness (*A_R_*) and private allelic richness (*PA_R_*) of each cohort were determined by implementing the rarefaction technique to correct for sampling bias in HP-Rare v1.1 [65]. Next, the diversity data was used to determine if it was normally distributed through a Shapiro–Wilk test, and a subsequent Kruskal–Wallis test was conducted to test for significant differences in diversity estimates between the six cohorts (*p* < 0.01) in XLSTAT [66]. Mean within-population pairwise relatedness (*r*) was also calculated in GenAlEx following the Queller and Goodnight method [67], with statistical significance (1000 permutations, 95% confidence intervals) determined for each population based on the two null hypotheses: (1) no differences from zero, and (2) no differences between populations.

The effective population size of each generation was calculated using the linkage disequilibrium (LD) method in NeEstimator v2.01, with a 95% confidence interval [68]. A random mating LD model was assumed and a minimum allele frequency of 0.02 was selected. A Wilcoxon signed rank test for heterozygote excess was performed in Bottleneck v1.2.02 [69] to test for evidence of a recent population bottleneck within each group. The analysis was composed of 1000 iterations, at a 5% nominal level. The two-phase model (TPM), which incorporates both the infinite alleles model (IAM) and the stepwise mutation model (SMM), was used. The TPM consisted of 30% IAM and 70% SMM, with a variance of 30. Wilcoxon tests were also conducted under the IAM and the SMM, for comparison.

To determine the level of genetic differentiation between the wild sampling populations and the mass-reared colony, as well as generational genetic differentiation across the temporal scale for the mass-reared colony, Arlequin v3.5.2.2 [70] was used to calculate pairwise *F_ST_* estimates between the six cohorts (10,000 permutations; *p* < 0.05). A hierarchical analysis of molecular variance (AMOVA; 10,000 permutations; *p* < 0.05) was also performed in Arlequin. Samples were separated into two groups: a wild group containing Wild_2015_ and Wild_2018_ and a mass-reared group containing generations F28, F48, F52 and F62 of the mass-reared population. To visualise the genetic differentiation between populations, a discriminant analysis of principal components (DAPC) was performed, using the R package *adegenet* [71]. Cross-validation was performed to determine the optimal number of principal components (PCs) to retain for the assignment of individuals to their genetic clusters.

Assessment of mating behaviour: Input file preparation and basic genotypic quality control were done as described above. Due to the small number of markers used, individuals with missing data at two or more loci were removed from the dataset. Genepop on the web v4.7 [62,63] was used to perform exact tests, to test for the conformation of loci to Hardy-Weinberg equilibrium (HWE; 10,000 dememorization, 500 batches, 5000 iterations per batch). The unbiased expected and observed heterozygosity (u*H_E_* and *H_O_*, respectively) and per locus *F_IS_* were calculated for three sampling populations: the candidate parents (F0), the offspring (F1) and the colony that the parents were sourced from (S). This was done in GenAlEx v6.503 [64]. These estimates were then compared between the three cohorts by performing a Kruskal–Wallis test (*p* < 0.01), to test for significant changes between the source population and offspring. The Queller and Goodnight method was then used to calculate the mean relatedness (*r*) in the source population and the candidate parent generation, as well as the individual pairwise relatedness estimates within each of the five parent pairs [67]. This was also performed in GenAlEx (1000 permutations, 95% confidence interval).

Each of the five families was tested for multiple paternity separately using two different methods: genotypic exclusion and full-pedigree likelihood. Vitassign v8.2.1 [72] was used for the genotypic exclusion method, while the full-pedigree likelihood method was performed in Colony v2.0.5.0 [73]. To implement the full-pedigree method in Colony, allele frequencies were calculated within each family. All markers were given a genotypic rate of 0.1 and a polygamous mating system was assumed for both sexes. Assuming monogamy for males, as well as assuming a population with or without inbreeding, yielded similar results.

## 3. Results

**Figure 1:**
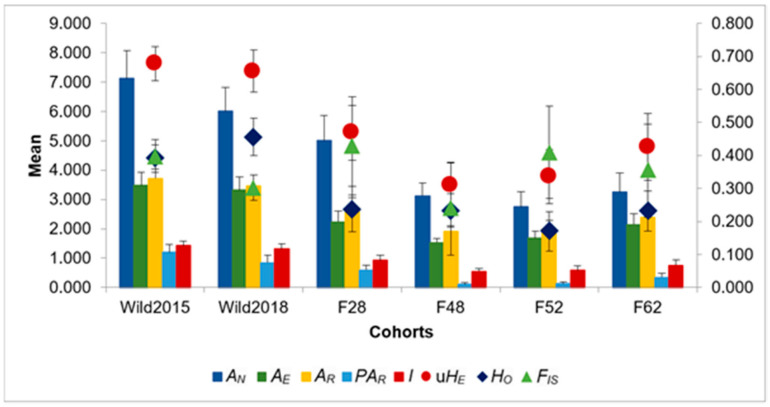
Summary of genetic diversity statistics across the six sampling populations. *A_N_*: number of alleles; *A_E_*: effective number of alleles; *A_R_*: allelic richness; *PA_R_*: private allelic richness; *I*: Shannon’s Index; u*H_E_*: unbiased expected heterozygosity; *H_O_*: observed heterozygosity; *F_IS_*: inbreeding coefficient. Error bars represent standard error (comprehensive results are given in Table S1).

**Figure 2:**
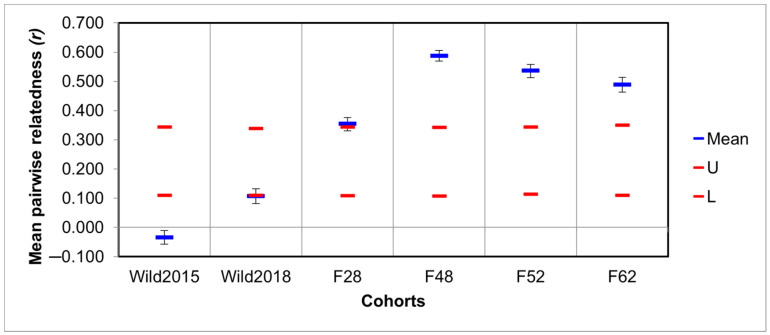
Mean within-population pairwise relatedness estimates, as calculated using the Queller & Goodnight method [67]. Blue bars represent mean relatedness, while red bars indicate the upper and lower 95% confidence intervals for the null-hypothesis of no significant differences in mean relatedness amongst groups. Error bars indicate the standard error for each mean.

Assessment of genetic diversity and differentiation: Based on the eight microsatellite markers, significant differences in all diversity estimates were found between the two wild cohorts and generations 48 and 52 of the mass-reared population (Kruskal–Wallis: *p* < 0.05). The number of alleles (*A_N_*), allelic richness (*A_R_*), and private allelic richness (*PA_R_*) of generation 62 also differed significantly from the two wild samples. The mass-reared colony suffered its greatest losses of genetic diversity within the first 52 generations, showing signs of a slight recovery in F62 (Figure 1). This was mirrored in relatedness coefficients, which increased dramatically in the mass-reared population over time (Figure 2). Inbreeding coefficients (*F_IS_*) showed a similar trend, with the exception of an abnormally low inbreeding coefficient in F48. All groups showed deviation from HWE. For a full list of results, see Supplementary Table S1.

Effective population sizes were low throughout all cohorts, with F28 and F48 of the mass reared colony estimated to have the largest effective population sizes (Table 1). Based on the two-phase model, these two cohorts also had significant heterozygote deficiencies, indicating recent population expansions.

**Figure 3:**
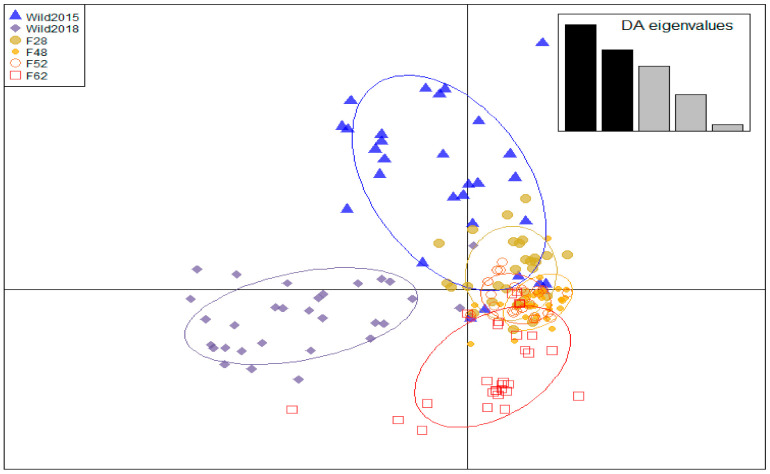
Discriminant analysis of principal components (DAPC) plot of six cohorts from two *Hermetia illucens* populations. Each cohort is represented by a unique shape and colour.

Based on *F_ST_* estimates, significant differentiation was found between all groups (*p* < 0.01; Table 2). The AMOVA also detected significant variation between the two populations and amongst the generations of each population (*p* < 0.01), as well as between individuals within each of the populations (*p* < 0.05; Table 3). Low to moderate differentiation was found between samples within the wild population and between the distinct generations of the mass-reared colony. Additionally, moderate to great differentiation was observed between the wild- and mass-reared populations. The discriminate analysis of principal components found that 29 principal components were needed for the optimum assignment of individuals to genetic clusters. The DAPC plot indicated that Wild_2018_ was the most distinct from all other groups, while the remaining five groups clustered together on the *y*-axis with varying degrees of overlap between them (Figure 3). The reduction in genetic diversity in the mass-reared population can also be seen in this plot.

**Figure 4:**
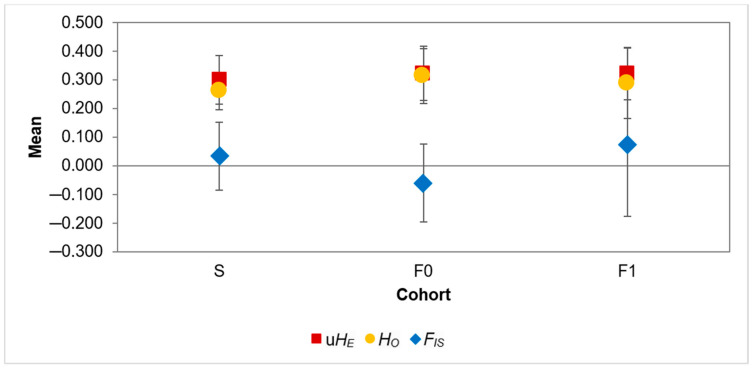
Inbreeding coefficients (*F_IS_*), unbiased expected heterozygosity (u*H_E_*) and observed heterozygosity (*H_O_*) for three *Hermetia illucens* cohorts: the source population (S), parent pairs (F0) and offspring (F1). Error bars indicate standard error.

Assessment of mating behaviour and parentage analysis: After removing poorly amplified and monomorphic markers, a panel of five markers was used to perform analyses. Differences in *F_IS_* estimates, unbiased expected and observed heterozygosity were all found to not be significant (Figure 4; Supplementary Table S2). However, the pairwise relatedness of each of the five individual parent pairs was greater than the mean pairwise relatedness of F0 as a whole (Table 4). Only one parent pair had a small inbreeding coefficient, caused by a novel mutation in the female fly that had only previously been observed in Wild_2018_. When the locus carrying this allele was removed, all parent pairs showed high levels of relatedness (Supplementary Table S3).

**Figure 5:**
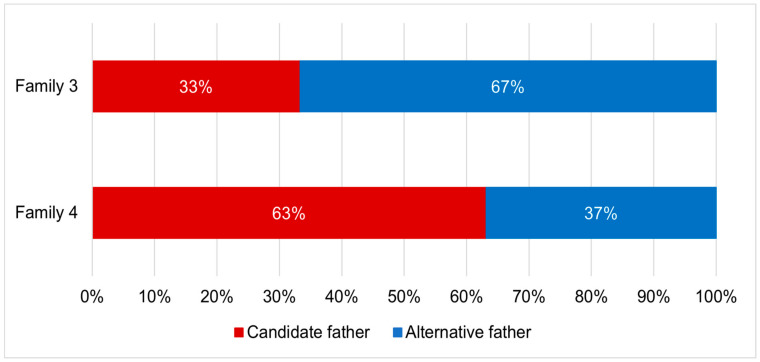
Relative contributions of both the candidate father and alternative father to the offspring in each of two *Hermetia illucens* families displaying multiple paternity, based on the genotypic exclusion method.

**Figure 6:**
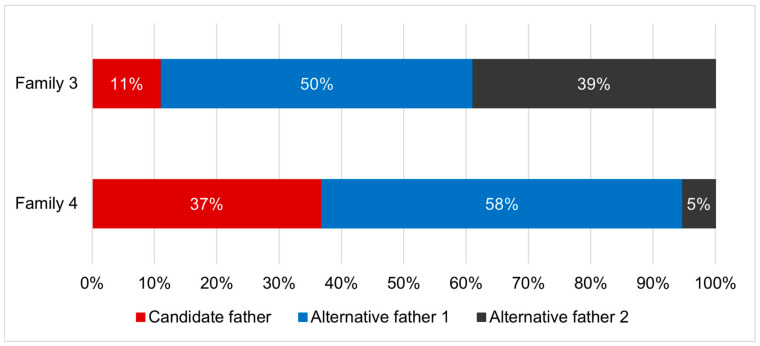
The relative contributions of the candidate father and two alternative fathers to the offspring in each of two *Hermetia illucens* families displaying multiple paternity, based on the full-pedigree likelihood method.

Parentage analyses found that two out of five tested families showed evidence for multiple paternity. The genotypic exclusion method identified two potential fathers per family (Figure 5), while the full-pedigree likelihood method identified three potential fathers per family (Figure 6).

## 4. Discussion

High genetic diversity was observed in the wild black soldier fly population, with both cohorts having large diversity estimates and showing low levels of relatedness (Figure 1 and Figure 2). Wild_2015_ had a significantly negative mean pairwise relatedness, and the cohort exhibited low observed heterozygosity compared to the expected heterozygosity (Figure 1), consistent with the Wahlund effect [74]. These findings suggest that Wild_2015_ was sampled from a recently admixed population of two genetically differentiated populations, which was further supported by the observation of isolate breaking when this cohort was allowed to interbreed in a study by Rhode et al. [11]. The apparent sampling of genetically distinct populations in Wild_2015_ may be a result of cryptic genetic structure in the wild BSF population. Park et al. [75] found significant local scale population differentiation in wild BSF in Korea. In conjunction with the high likelihood of local extinction and recolonisation events [76,77], caused by the limited availability of resources encouraging localised migration by wild females, this could explain the observation [75,77]. This may also have led to the inflation of the inbreeding coefficient in the Wild_2015_ cohort, as *F_IS_* estimates are based on estimates of heterozygosity [78]. Furthermore, overlapping generations add an additional layer of structure to wild populations. This is particularly relevant if temporal genetic differentiation occurs, as observed in the current study with the Wild_2018_ cohort showing moderate differentiation (*F_ST_* estimates) and clustering separately on the DAPC plot from the Wild_2015_ cohort (Table 2 and Figure 3) [79]. Low estimates of effective population size in the two wild cohorts (Table 1), in spite of their high levels of genetic diversity, are a further indication of population structure in the wild population, likely due to stochastic effects of pronounced random genetic drift [79,80,81,82,83].

The wild population seems to be characterised by an abundance of genetic diversity. In contrast, the mass-reared colony has been marked by a clear loss of diversity over time (Figure 1). This pattern of diversity loss in domesticated populations has previously been observed in various captive fly populations from several different species [25,84,85]. The three latter generations of the mass-reared colony showed signs of significant inbreeding, with relatedness coefficients ranging from 0.49 to 0.59 (Figure 2). These values are comparable with the relatedness coefficient of 0.5 associated with full siblings [86]. The level of relatedness in F48 was so high that its expected heterozygosity was underestimated (Figure 1 and Figure 2). This phenomenon has previously been studied by Harris and DeGiorgio [87]. As *F_IS_* estimates are affected by estimates of heterozygosity [78], the high degree of inbreeding in this generation was not reflected in its estimated inbreeding coefficient (Figure 1).

Interestingly, F28 and F48 were estimated to have the largest effective population sizes of all groups (Table 1). Although effective population sizes may have been underestimated in the wild population [79,81,82], a controlled environment optimised for reproduction may also boost effective population size within the mass-reared colony, where the absence of fluctuating stressors (that are containing in the wild) are conducive to high reproductive output. This can result in high mutation- and recombination rates, transiently restoring some lost diversity [88,89,90]. Supporting this, the Wilcoxon signed rank tests found significant heterozygote deficiencies in these two generations. Heterozygote deficiencies indicate potential expansions in the mass-reared colony during this time, which can be associated with increased production outputs (Table 1). However, as no new individuals were introduced into the population, genetic diversity continued to decline. Had the population been augmented with immigrant flies, a lasting increase in genetic diversity and effective population size would have been observed [30]. Furthermore, even though F28 and F48 had the greatest effective population sizes, effective populations of less than 500 are at risk of fitness loss, while effective populations of less than 100 are at risk of inbreeding depression [91]. As the maximum estimated effective population size was 59 and genetic diversity in later generations of the mass-reared colony was found to be low, augmentation with immigrants from the wild population could be considered to introduce genetic diversity into the mass-reared colony [29].

However, to reduce the risks associated with population augmentation in future genetic management and breeding strategies, immigrants need to be sourced from a population that is genetically similar to the receptor population. Flies that are too differentiated from the mass-reared colony may struggle to adapt to the artificial environment, potentially causing a loss of fitness rather than the desired increase, a phenomenon known as outbreeding depression [29,30,92]. Significant differentiation was found between the wild- and mass-reared populations, with the AMOVA finding greater differentiation between the two colonies than between generations within each colony (Table 3). Further, *F_ST_* estimates indicated moderate differentiation between the mass-reared colony and Wild_2015_, but great differentiation between the mass-reared colony and Wild_2018_ (Figure 2). Wild_2018_ also clustered separately from all other groups on the DAPC plot (Figure 3). Differentiation between the wild- and mass-reared colonies therefore appears to be increasing over time. Temporal structure in the wild black soldier fly colony, as well as random genetic drift and potentially novel selection regimes in the mass-reared population, are contributing to this increased differentiation. Greater differentiation between the wild- and mass-reared colonies may influence the potential success of population augmentation of the mass-reared colony with wild flies. It would thus be advisable to test the potential success of population augmentation on a small subset of the mass-reared colony before introducing wild immigrants into the general population, as has previously been done in *Drosophila* [27].

To gain a more comprehensive understanding of the factors contributing to the maintenance of genetic diversity in a BSF colony, the mating behaviour of individuals was evaluated on a basic population level, testing for assortative mating by comparing genetic relationships within and between generation 48 of the mass-reared colony (S), a subgroup containing five parent pairs (F0) and their offspring (F1). A lack of significant differences in diversity estimates between the three groups (Figure 4), provided initial evidence for random mating within the mass-reared population. However, three of the five markers deviated from Hardy-Weinberg equilibrium in F1, suggesting assortative mating might be occurring. Although the mean relatedness amongst all possible parent pairs was comparable to the mean relatedness of the source population, individual parent mate pairs displayed high levels of relatedness indicating a mate preference for genetic similarity, i.e., positive assortative mating (Table 4) [48,93]. As this increased relatedness was found using loci from random genomic regions, as opposed to genes associated with known phenotypes, the phenotypic mechanism underlying this positive assortative mating cannot be determined at this point. Furthermore, the occurrence of positive assortative mating in F0 as a consequence of inbreeding in the mass-reared population cannot be excluded [49,50]. However, as inbreeding increases genome-wide linkage disequilibrium, mate selection for desirable traits could potentially be detected in regions not directly associated to a trait [94].

Evidence for multiple paternity was found in two out of five families, with the genotypic exclusion method estimating two contributing fathers per family and the full-pedigree likelihood method estimating three. These findings provide evidence for the presence of polyandry in the species. Polyandry has previously been observed in various lekking insects, including fruit flies and moths [95,96]. The absence of parental care in these species lends itself to having more energy available for mating in females, increasing the likelihood of multiple mating [36]. Additionally, the mass-rearing environment could be conducive to multiple mating, through controlling factors such as temperature, light intensity and cage density [89,90]. High levels of relatedness between flies from this population may also contribute to an increased female reproductive life span. Male *Drosophila* flies have previously been found to be less competitive when closely- related and reared together. Less harm was caused to females during mating, increasing both mating success and their reproductive life span [97]. Thus, it remains to be investigated if polygamous mating is a “specialised” behaviour in the captive environment or whether it is pervasive amongst wild populations as well.

The occurrence of polyandry and resulting multiple paternity provides evidence that adult flies can mate multiple times with genetic consequences for the offspring, despite not being able to replenish energy between mating events. Thus, disproving the currently held hypothesis that although multiple mating has been observed in BSF as a behaviour, the energetic constraints on gametogenesis effectively render the species genetically monogamous. Polygyny is therefore also possible for this species but needs to be assessed in the future. Some lekking species display polygyny through the emergence of males with a fixed number of sperm, which is then divided amongst partners. Ejaculate size decreases with each successive mating event until sperm and energy reserves are depleted [46,95].

The occurrence of polyandry in the mass-reared colony has positive implications for its genetic management. Polyandrous mating allows for greater genetic diversity in offspring than monogamous mating, thereby aiding both the maintenance of genetic diversity and the reintroduction of genetic diversity into homogenous populations [39,98].

## 5. Conclusions

The black soldier fly has shown particular promise as both a bioremedial agent and a source of usable animal protein and other bio-products, which has led to the establishment of large industrial production facilities. As domestication progress, particular emphasis is being placed on genetic management and selective breeding for the enhancement of production characteristics in commercial populations. However, unlike conventional livestock, insects, including the BSF, have very different life history characteristics that might prove challenging for conventional animal breeding methods. In particular, *r*-selected life history characteristics predispose the species to a rapid loss of genetic diversity that might hinder population fitness, productivity and future enhancement potential, as has been demonstrated in this study with a significant loss in genetic diversity in a mass-reared BSF population over the long-term. Furthermore, other than the classical microevolutionary processes associated with small and isolated populations, genetic diversity is also influenced by the dynamics of mate choice and breeding behaviour. The first evidence of positive assortative mating and multiple paternity for the BSF is presented here. The genetic management of BSF colonies will thus entail a careful interplay between managing colony demographic trajectories, mating systems, and selective breeding.

## Figures and Tables

**Table 1:** Wilcoxon signed rank test *p*-values for heterozygote excess and deficiency under the infinite alleles model (IAM), stepwise mutation model (SMM) and two-phase model (TPM), as well as estimates of effective population size (*N_e_*) calculated using the linkage disequilibrium method. 95% confidence intervals are given in brackets.

Parameter	Wild_2015_	Wild_2018_	F28	F48	F52	F62
**Sample size (*n*)**	30	30	30	30	30	29
***N_e_***	22.1	30.0	59.0	56.7	22.6	24.4
	(14.7–36.6)	(16.4–82.4)	(21.7–∞)	(14.9–∞)	(5.3–∞)	(7.4–∞)
**Wilcoxon test**						
**IAM**						
*H_E_* excess	0.230	0.004 **	0.680	0.727	0.148	0.039 *
*H_E_* deficiency	0.809	0.998	0.371	0.320	0.945	0.973
**SMM**						
*H_E_* excess	0.902	0.986	1.000	0.986	0.852	0.711
*H_E_* deficiency	0.125	0.020 *	0.002 **	0.020 *	0.188	0.344
**TPM**						
*H_E_* excess	0.727	0.371	0.990	0.973	0.406	0.289
*H_E_* deficiency	0.320	0.680	0.014 *	0.037 *	0.656	0.766

* indicates statistical significance at the 5% nominal level. ** indicates significance at the 1% nominal level.

**Table 2:** Pairwise *F_ST_* estimates for wild and mass-reared cohorts of *Hermetia illucens*.

	Wild_2015_	Wild_2018_	F28	F48	F52
**Wild_2015_**					
**Wild_2018_**	0.062 **				
**F28**	0.094 **	0.162 **			
**F48**	0.139 **	0.225 **	0.112 **		
**F52**	0.096 **	0.172 **	0.033 **	0.047 **	
**F62**	0.160 **	0.163 **	0.082 **	0.201 **	0.103 **

** indicates significance at the 1% nominal level.

**Table 3:** Hierarchical AMOVA of *Hermetia illucens* based on eight microsatellite markers. The wild and mass-reared populations were grouped separately.

Source of Variation	Variation (%)	Fixation Index
Amongst groups	6.87	*F_ST_* = 0.150 **
Amongst generations within groups	8.10	*F_SC_* = 0.087 **
Within groups	85.03	*F_CT_* = 0.069 *

* indicates statistical significance at the 5% nominal level. ** indicates significance at the 1% nominal level.

**Table 4:** Mean pairwise relatedness of a *Hermetia illucens* population (S) and a group of candidate parents from the population (F0), as well as pairwise relatedness estimates for each of the five individual parent pairs. Standard errors for the two sample groups are indicated in brackets.

**Population**	**Mean Pairwise Relatedness**
Source population (S)	−0.034 (−0.094–0.126)
Candidate parents (F0)	0.093 (−0.421–0.347)
**Parent Pair**	**Pairwise Relatedness**
Family 1	0.455
Family 2	0.455
Family 3	1.000
Family 4	−0.430
Family 5	0.700

## Data Availability

All data are captured in the main text or as supplementary materials.

## Supplementary Materials

The following are available online at https://www.mdpi.com/article/10.3390/insects12060480/s1, Table S1: Genetic diversity indices per microsatellite marker for *Hermetia illucens* populations across six generational time points: polymorphic information content (*PIC*); average number of alleles (*A_N_*); effective number of alleles (*A_E_*); rarefied allelic richness (*A_R_*); private allelic richness (*PA_R_*); Shannon’s information index (*I*); observed heterozygosity (*H_O_*); unbiased expected heterozygosity (*uH_E_*); fixation index/inbreeding coefficient (*F_IS_*); and null allele frequencies (*Fr_(Null)_*), as well as the standard error (*SE*) for each mean estimate. An asterisk (*) indicates deviation from Hardy-Weinberg equilibrium (*p* < 0.01), Table S2: Per locus estimates of unbiased expected heterozygosity (*uH_E_*), observed heterozygosity (*H_O_*) and inbreeding coefficients (*F_IS_*) across three cohorts of *Hermetia illucens*, as well as the standard error (*SE*) for each mean estimate. An asterisk (*) indicates deviation from Hardy-Weinberg equilibrium (*p* < 0.01), Table S3: Mean pairwise relatedness of the source population and candidate parents, as well as pairwise relatedness estimates for each of the five parent pairs, with the exclusion of the locus Hill_23. Standard errors for the two sample groups are indicated in brackets.
